# Assessment of users’ behavior in Lithuanian online communities

**DOI:** 10.3389/fpsyg.2023.1265341

**Published:** 2023-10-03

**Authors:** Aelita Skarzauskiene, Monika Mačiulienė

**Affiliations:** Faculty of Creative Industries, Vilnius Gediminas Technical University, Vilnius, Lithuania

**Keywords:** online communication, virtual communities, social technologies, quantitative survey, Internet

## Abstract

**Introduction:**

Online communities are gaining importance in modern society by actively structuring public opinion and initiating discussions about various socio-economical issues. As information and communication technologies advance, the online communities are confronted with novel technological and societal hurdles (the spread of misinformation, lack of active participation). To boost their efficacy and productivity, it’s crucial to enhance our understanding of user behavior, communication avenues and potential future trends of development.

**Objective:**

Online platforms serve a function beyond simply sharing information or knowledge; they act as influential social networks affecting various societal sectors, including politics, culture and the economy. There is a need to recognize online communities not as static entities, but as dynamic, evolving systems of collective intelligence.

**Methods:**

A representative quantitative study was carried out between 1 to 30 of October 2022 through direct, in-person interviews conducted at the respondent’s residence (known as an Omnibus survey). The sample of respondents is representative of the entire population of Lithuania regarding essential socio-demographic characteristics. By thoroughly analyzing data gathered from a comprehensive quantitative study, the study raises awareness of the issues surrounding online communities, while also shedding light on social networking behavior within these virtual spaces.

**Discussion:**

Despite the multitude of challenges inherent to virtual communication, there remains a significant knowledge gap in comprehending general user behavior within these online communities. The current research aims to bridge this gap by investigating user behavior in Lithuanian online communities.

## 1. Introduction

Communities are integral parts of human societies, comprising individuals with shared interests, views or objectives. An online community takes this notion into the digital realm, where it describes a group of individuals united around a common purpose, interest or goal on the Internet. Different researchers have explored this concept through varying lenses. Some focus on the technological interaction between members (Herbert, 2012; Abedin et al., 2019; Chen, 2022) while others emphasize the social value created within these communities (Sproull and Arriaga, 2007; Lykourentzou et al., 2011). Despite differences in the medium of communication or the specific objectives, all online communities share common features, such as the need for active participants pursuing individual goals that ultimately contribute to the communal objective (Lykourentzou et al., 2011).

The developments of both the digital technologies and how society uses such tools led to significant transformations in how the online communities function. Initially, these platforms primarily served as casual outlets for individuals to share unofficial information, personal concerns, or rumors. Such communities were often seen as secondary spaces to traditional forms of communication and not taken seriously in many instances. Over time, the nature of online communities started changing with the rise of Web 2.0 and social networking sites, enabling more dynamic interactions. As such platforms gained popularity and credibility, governments and corporations began recognizing the potential of online communication. In this regard, online communities have increasingly played pivotal roles in structuring public opinion and instigating discussions about various socio-economic issues (Chua et al., 2007; Xu and Zhang, 2009). As we venture further into the era of Web 3.0, the role of artificial intelligence, virtual reality and other advanced technologies in shaping online communities becomes an exciting frontier to explore (Thomas et al., 2020).

Despite the recognized advantages they offer, online communities face a plethora of challenges that cannot be overlooked. One of the critical criticisms of virtual networks is the limited physical contact between participants, leading to a loss of interpersonal connection that is inherent in face-to-face interactions (Abedin et al., 2019; Guo and Fussell, 2022). Furthermore, the safety and security of online communities can be compromised, with concerns ranging from cyber frauds to online harassment, hazing, and the violation of privacy (Horrigan, 2001; Mansour and Francke, 2021). The reliability and quality of information within these communities are also questionable, given the freedom and anonymity these platforms provide. Misinformation can spread rapidly, leading to confusion and potential harm (Lăzăroiu and Adams, 2020; Bizzotto et al., 2022; Zhen et al., 2023). Furthermore, the imbalance of participation in many online communities raises another issue; a small fraction of users often contributes the most content, while the majority engage passively or lurk, adding little to the conversations (Debaere et al., 2018; Fullwood et al., 2019).

Another research question is related to deeper understanding of the motivations driving users to participate in online communities. Factors such as enhancing user engagement, improving online safety, increasing information reliability, and promoting respectful communication can be addressed more effectively by comprehending what compels users to interact within these communities. This knowledge can also aid community developers in devising strategies that maximize customer engagement while minimizing potential negative impacts. Many studies have concentrated on the motivations behind individuals’ active participation in online communities (Bateman et al., 2011; Ehrlich et al., 2014). These motivations range from the desire for recognition from community managers and fellow members (Ardichvili et al., 2003; Füller et al., 2007), to the opportunity to edit and update content, contributing to the collective knowledge of the community (Wagner et al., 2014). The rich communication environment of online communities is well-documented, demonstrating the behavioral possibilities that surpass those in previous computer-mediated communication contexts (Wise et al., 2012; Wagner et al., 2014). For example, content generated in these communities tends to be publicly visible and enduring, facilitating lasting associations between members and content (Baek and Kim, 2015). Despite the extensive research on why individuals choose to participate in online communities (Imroz, 2019; Chen, 2022), less attention has been paid to the factors that discourage interaction (Yadav et al., 2023). In this regard, many studies overlook the complexities and variations of these motivations across different community types and demographics. There is a need to recognize online communities not as static entities, but as dynamic, evolving systems of collective intelligence. The possibility to include large numbers of diverse participants into online collaboration ensures the emergence of greater intellectual capabilities. In online communities not only humans, but also information communication technologies (ICT) are involved in knowledge creation or aggregation. Online platforms serve a function beyond simply sharing information or knowledge; they act as influential social networks impacting various societal sectors, including politics, culture and the economy. Hence, as the online communities develop, the important research question is how to address these challenges effectively, whether through improved ICT and platform design, community guidelines or legislative measures. Despite the multitude of challenges inherent to virtual communication, there remains a significant knowledge gap in comprehending general user behavior within these online communities. Online behaviors are often culture and country specific, so this study aims to bridge this gap by investigating user behavior in Lithuanian online communities. The next chapter provides deeper insights about the socio-cultural context of online communities in Lithuania. The identification of peculiarities and trends in users` behavior from former Soviet countries creates additional value to this paper and originality to the research. The third chapter is dedicated to describe quantitative research methodology and results of representative survey of Lithuanian population with the special focus on online communication and participation in online communities.

## 2. Challenges of online communities in Lithuania

Lithuania’s online communities offer a unique research platform for studying the potential of networked structures. With a solid foundation of information technology infrastructure, impressive Internet accessibility and a highly-educated population, Lithuania is well-positioned to evolve into an ideal networked society. Lithuania’s long-term plans reinforce this digital readiness, with the 2030 National Progress Strategy emphasizing the importance of a sound information and digital technology infrastructure. Additional strategic measures include the 2020–2030 Lithuanian Industry Digitalisation Roadmap and the National Cybersecurity Strategy for 2018–2023.

However, a paradox emerges when considering Lithuania’s civic engagement. Despite high digital access, the annual Lithuanian Civic Empowerment Index (Civil Society Institute, 2021) suggests that active participation remains an issue. In 2020, the Index reached its peak of 41.3 out of 100, driven by increased donations, local community involvement, and public dialogs. Yet, a representative survey revealed that over half of the respondents (53.8%) neither participated in voluntary activities nor intended to. Most existing volunteers (41.5%) participate sporadically. This lack of consistent civic engagement raises questions about the full realization of Lithuania’s networked society potential. Lithuania’s digital societal integration is measured by the EU Digital Economy and Society Index (European Commission, 2022). With a 2022 score of 53.9, exceeding the EU average of 52.6, Lithuania was ranked 14th amongst EU countries. The index includes indicators such as e-connectivity access, societal digital skills, e-resource usage, digital technology application in business, and the level of digital public services. Lithuania made strides in several areas, including digital public services, electronic information sharing, and Internet service usage by citizens. However, the digital skillset of the Lithuanian public scored lower than the EU average, signifying an area for improvement.

The development of Lithuania’s online communities has been influenced by historical factors. The soviet occupation negatively affected non-governmental organizations, stifling civil activity (Marček, 2008). As result, independent Lithuania grapples with revitalizing civic engagement through rebuilding national identity and trust (Kėrytė, 2010). Nonetheless, existing online communities demonstrate a certain level of social maturity, leaning toward social and business innovation (Skaržauskienė and Mačiulienė, 2016). Lithuanian local communities hold considerable innovative potential, tackling issues specific to their environment. Yet, research reveals that Lithuanians typically seek self-actualization or leisure rather than active public interest representation (Tijunaitiene and Bersenaitė, 2013). The absence of a unified statistical database of online community accounting further compounds the issue. Our understanding of user behavior in online communities is limited, and Lithuania, with its unique historical and societal context, provides a fertile ground for investigation. By implementing representative survey of Lithuanian population, this study provides “helicopter view” on online activities in Lithuania and enhance general understanding of online communication and social networking behavior.

## 3. Materials and method: quantitative survey on users’ behavior in online communities in Lithuania

The survey was carried out between 1 to 30 of October 2022. A representative quantitative study was carried out through direct, in-person interviews conducted at the respondent’s residence (known as an Omnibus survey), with the aid of computers (Computer Supported Personal Interviews). For these computer-facilitated surveys (CAPI), the survey tool – a pre-programmed questionnaire – was used. This software allowed automatic regulation of quotas, logic and typing errors, logical associations between queries, the progression of questions, and their responses. Interviewers ensured that the order of the questions remained consistent throughout. Data were stored directly in a digital format, reducing the potential for human error during data entry. A randomized multi-stage sampling method was employed to select the households and respondents to be included in the survey. The sample of respondents is representative of the entire population of Lithuania regarding essential socio-demographic characteristics (using random stratified sampling, taking into account specific criteria related to participation in online communities). The target group in this study is the Lithuanian population aged 15–74. The household surveys (from which respondents will be selected) are selected in the following steps:

- Geographic distribution of the sample. Geographically, the sample proportions correspond to the overall population proportions in the different regions. The most recent data from the Statistical Office of the Republic of Lithuania are used;
- Randomly selecting the initial sampling points, i.e., the addresses from which the sampling route starts, from the Population Register of the Lithuanian Statistical Department database. A total of 117 initial sampling points were used;
- Households were selected using a route sampling approach. Every second apartment/house in an urban or rural area was visited starting from the initial sampling point. Respondents are selected using the ‘youngest male’ sampling rule.

In total, 1,135 respondents were surveyed (564 selected persons refused to take part in the interview; 68 selected persons did not meet the criteria for the survey, for example, did not use the Internet at all; 6 questionnaires were canceled).

### 3.1. Quality control: reliability and validity

The reliability of survey results depends to a large extent on the way the survey is conducted by the data collectors and on whether all quality control standards for data collection are met. To guarantee the validity and reliability of the gathered data, an independent Quality Control Unit was established. This unit’s role includes overseeing data quality and coding responses to open-ended questions. The data quality check included a check and assessment of compliance with the household selection rules, the respondent selection rules, the quality of the questionnaire, the quality of the route or other respondent record sheet, the general principles of the survey, and the interviewer’s behavior during the survey. Standardized verification techniques were used to check the quality of the data: (1) physical verification of the data series, (2) telephone verification, (3) verification of compliance with the rules of the methodology during the survey, and (4) on-site verification of the conduct of the interview. In addition, the reliability of the questionnaire was tested using test–retest reliability method, which evaluates the consistency of a measure across the time (Berchtold, 2016).

Data analysis was carried out using SPSS/PC software. The *χ*^2^ test was used to calculate the dependence of one criterion on the other. A relationship was considered statistically significant if the *χ*^2^ criterion statistic (*p*-value) is less than or equal to 0.05 (*p* < =0.05); as a result, it can be said with 95% confidence that the values of the answers to one question are statistically significantly correlated with the values of the answers to the other question. When assessing the results, it will be necessary to pay attention to the statistical bias that arises from the fact that a sample of respondents is selected rather than from a continuous survey. This error is calculated mathematically. The maximum statistical error in the estimation of the responses of all respondents (1,000 people) is due to sampling. It is equal to ±3% at the 95% confidence level, i.e., it will not exceed ±5% at the 95% confidence level.

All demographic data was collected adhering to the General Data Protection Regulation (GDPR) stipulated by the European Union, and was utilized exclusively for research and statistical purposes. Furthermore, to take part in the survey, all research participants were required to complete a consent form. This was incorporated into the introductory segment of the survey questionnaire, ensuring that participants understood and agreed to the data collection process.

## 4. Empirical survey results IN Lithuania

The questionnaire is based on the following constructs and their scales:

I. Internet usage frequency: daily, once a week, several times a week, several times a month, once in three month, not using.
II. Extent of involvement in online communities, motivation, and participation character/ activeness: *frequency of involvement, most frequent online activities, motivation for engagement in online communities, reasons for not participating in online communities*.
III. Extent of involvement of online communities focusing on social issues and civic initiatives motivation, and participation character/ activeness: *frequency of involvement, motivation for engagement, areas of interest in online communities focused on societal issues, reasons for not participating.*
IV. Quality evaluation of different aspects of online communication: *level of satisfaction, benefits and disadvantages of online communication*.

The validity of measurement was estimated through expert judgment using on content validity method. Content validity evaluates the extent to which the measurement covers all aspects of the concept measured, and how well an instrument covers all relevant parts of the construct (Sireci, 1998). This method was chosen because of exploratory synoptic nature of the research conducted. Additionally, there was certain degree of general agreement among the experts, about what the particular construct represent.

### 4.1. Internet usage frequency

Approximately 72% of respondents of the survey access the Internet at least weekly, while nearly one-third of Lithuanians between the ages of 15 and 74 either use the Internet less frequently than quarterly or not at all. Daily Internet usage is reported by 53% of respondents. Among Internet users, 63% (equivalent to 41% of the total population) frequent various online community websites. Of these Internet users, one-third (35%) visit online communities daily, and a fifth (21%) visit at least once a week. However, over a third of Internet users express no interest in online communities – the majority of them never visit these sites, and 6% have accounts but do not utilize them. Daily participation in online communities is notably higher among Internet users aged between 15 and 29, individuals with primary and secondary education, students, schoolchildren, and single individuals. Those aged between 30 and 39 are more prone to use these sites weekly or less frequently.

### 4.2. Extent of involvement in online communities, motivation, and participation character/activeness

**Figure 1:**
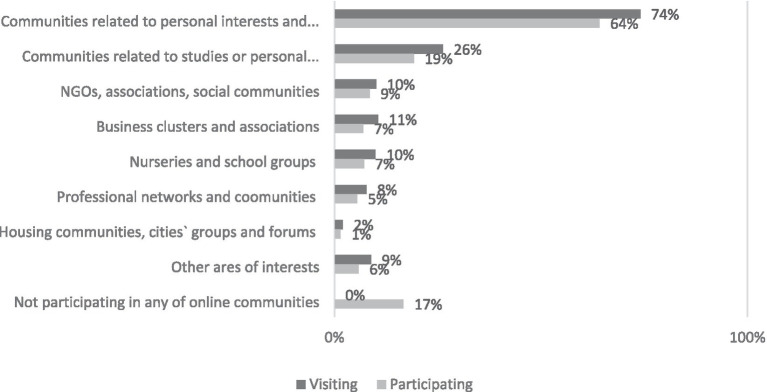
What type of online communities are you interested in (more than one choices are possible)? Percentage of respondents who visit different sites of online communities (*N* = 514).

Participation in online communities (not only visiting, being interested and gathering information, but also creating content such as writing, commenting and sharing experiences) is exclusively related to the personal interests of the participant, who usually chooses communities groups or forums relevant to his lifestyle, hobbies, and interests (74% visit, 64% participate). The second most popular topic is communities related to studies/scholarship (26% visit, 19% participate) (see Figure 1). Pages related to personal interests and hobbies are statistically significantly more frequently visited (used) by digital visitors aged 40–49, those with secondary education, and those with an income of more than 900 EUR. Pages related to studies or education are more frequently visited by users aged 15–29, students, schoolchildren, single people, and those who visit online communities’ websites daily. When it comes to involvement in online communities, highly educated women, along with those who are divorced or widowed, are more likely to participate in groups associated with personal lifestyles, interests, and hobbies. Individuals living in households with two or more children show a greater likelihood of engaging in online communities linked to academic pursuits or education.

**Figure 2:**
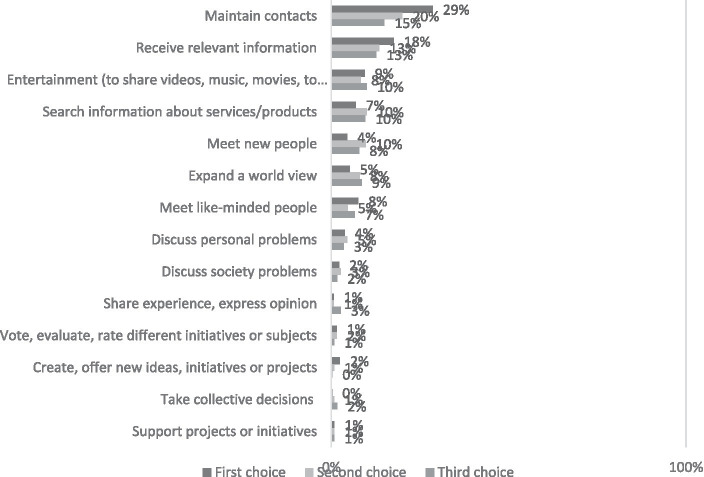
What is your motivation to visit sites of online communities? Percentage of respondents who visit different sites of online communities (*N* = 514).

The primary reasons for visiting online communities include maintaining social connections and seeking information on pertinent topics, with 64 and 44% of all responses, respectively. Online communities also serve as platforms for entertainment (28% of responses) and for finding information about products and services (26% of responses). They further facilitate contributions to ideas, initiatives, or projects initiated by others, decision-making processes (like voting for initiatives or selecting charitable causes), and the creation and proposition of new ideas, initiatives, or projects (1–3% of mentions). Single individuals and those living in two-person households are more inclined to participate in online communities to sustain social connections and to remain in contact with acquaintances. Residents of the three largest cities show a higher likelihood of utilizing these platforms to seek information on matters of concern (as detailed in Figure 2).

**Figure 3:**
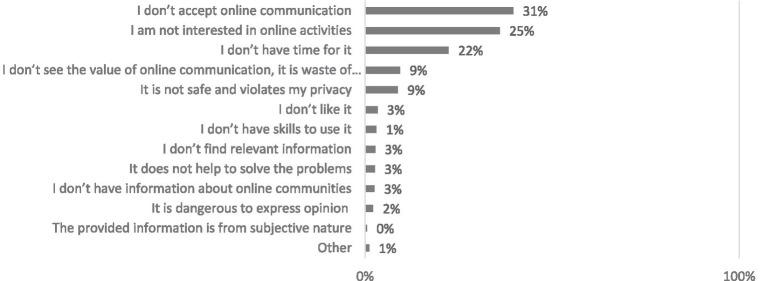
What are the reasons for not participating in online communities? Percentage of respondents who do not visit sites of online communities (*N* = 303).

Not being comfortable with online communication, not being interested in discussions, or lack of time are the most common reasons for not participating in online communities or visiting their websites. Internet users with one child and those living in cities other than the most prominent Lithuanian cities were likelier to say that they do not find this way of communicating acceptable. Those aged 30–39 and residents of the three biggest cities were likelier to say they were not interested in using virtual communities and social networking websites. At the same time, those with two or more children emphasized the lack of time (see Figure 3).

### 4.3. Extent of involvement of online communities focusing on social issues and civic initiatives motivation, and participation character/activeness

**Figure 4:**
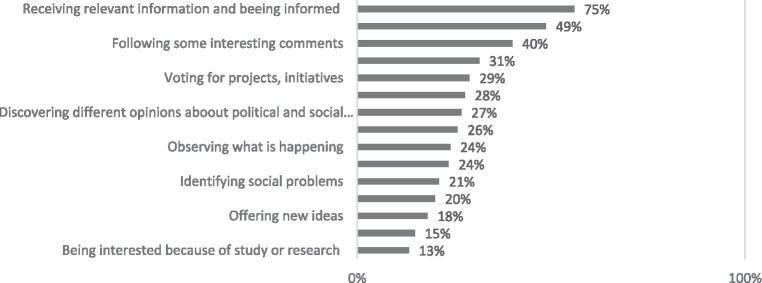
What is your motivation to visit sites of online communities focused on societal issues? Percentage of respondents who visit online communities focused on societal issues (*N* = 147).

A mere 18% (equating to 12% of the total population) of all Internet users frequent online community websites centerd around public issues. Users who have one child in their household and those who participate in online community activities daily are statistically more likely to use websites focusing on societal matters. Based on the findings, online communities offer participants pertinent information, intriguing articles, commentary, and overall, expand their worldviews. Nearly half of the visitors to these sites also take the opportunity to express their viewpoints. Married and cohabitating individuals are significantly more likely to contribute to initiated projects by suggesting possible improvements and applying their knowledge to identify and solve various social issues. On the other hand, single individuals tend to seek like-minded people. Women show a higher likelihood of seeking relevant information, and those with one child in their household are more inclined towards reading articles and comments (as depicted in Figure 4).

**Figure 5:**
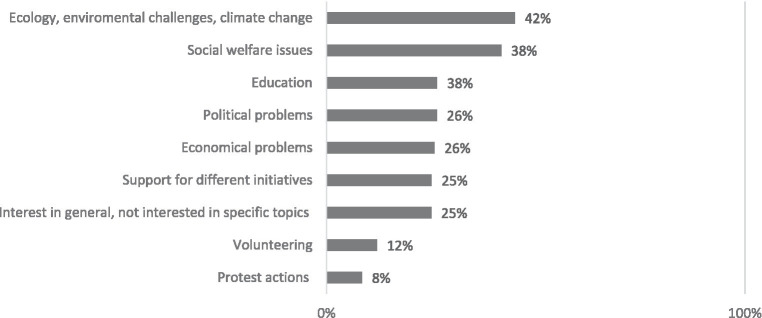
What topics, initiatives, and areas of discussion are relevant for you? Percentage of respondents who visit online communities focused on societal issues (*N* = 147).

Visitors of online communities focused on societal issues are most interested in ecology, environmental challenges, and climate change, as well as in education and social welfare topics. Education issues are more frequently highlighted by people with households of three people and those who visit virtual communities/social networking websites daily. Almost half of the respondents claim that almost all areas of interest are covered by different online communities. They do not lack websites, virtual communities, or initiatives (see Figure 5).

**Figure 6:**
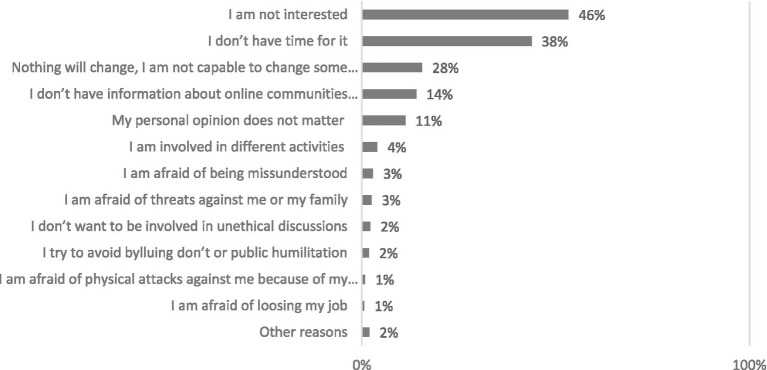
Why do you not participate in social problem-solving-oriented virtual activities? Percentage of respondents who use the Internet but do not participate in social problem-solving-oriented virtual activities (*N* = 670).

Internet users who are either unaware of, or refrain from participating in, online activities centerd on addressing societal issues, often cite a lack of interest or insufficient time as their primary reasons. A minimal fraction of these individuals (1–3%) express concerns over participating in virtual activities aimed at social problem-solving due to perceived dangers – such as fear of being accused of ulterior motives, threats, harassment, potential physical harm, or job loss. However, respondents underlined the importance of state oversight of Internet content and called for increased focus on data security and intellectual property protection. Single women are more likely to abstain from participation due to a perceived lack of appeal. Those claiming a lack of time are more likely to be aged 50–59 and to have two or more children (as illustrated in Figure 6).

### 4.4. Quality evaluation of different aspects of online communication

**Figure 7:**
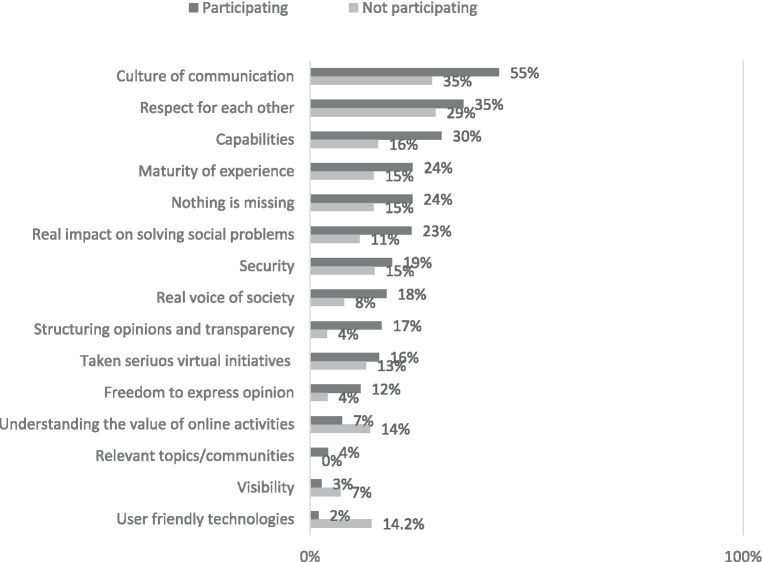
What important aspects are missing in solving social problems through online communication? Percentage of respondents who use the Internet (*N* = 817).

Participants of online communities generally express a high level of satisfaction with virtual communication for discussing or resolving socio-political issues, boasting an average satisfaction score of 3.8 out of 5. Those with higher educational attainment are more likely to find satisfaction in digital communication and the use of social technologies. However, both engaged and non-engaged groups in online communities share a common need for more respect, a cultivated communication culture, improved digital skills, and enhanced user experience. Notably, active users express a greater desire for meaningful influence on socio-political issues, a genuine societal voice, clear topic structure, freedom of expression, and convenient technological solutions. Opinions largely align across different socio-demographic groups of users (Figure 7).

Internet users generally hold a favorable view of online communication, with the lowest average score being 3.4 out of 5. Aspects that are particularly appreciated include the ability to find like-minded individuals, acquire knowledge, obtain relevant information, voice opinions, and propose new ideas and initiatives. These top five features are often emphasized by Internet users aged between 20 and 29, students, school children, single individuals, and consistent Internet users. The majority of Internet users (over 70%) concur that online communities should bear strict liability for any infringements of others’ rights. They also believe that site administrators should be held accountable for disseminated content, that these administrators should scrutinize the information they share, and that virtual community activities should fall under detailed legal regulation. Most Internet users acknowledge the issue of identity theft in the virtual world, such as the creation of fraudulent profiles or blogs impersonating renowned individuals. They also agree that there is a greater prevalence of intolerance and defamation online than in real life.

## 5. Discussion

The vast majority of surveyed Internet users (63%) frequent these online communities, making them an ideal platform for civic engagement and interaction. The study also reveals that the level of participation in these communities is determined largely by personal interests. Online communities centerd around hobbies, lifestyle, and personal interests attract the highest levels of active participation. This active participation extends beyond passive engagement, with members actively contributing to content creation, discussion, and experience sharing. It suggests that online communities can be more effectively leveraged for civic participation by aligning them with people’s interests, thereby encouraging greater active involvement. The findings also indicate that there is a high level of engagement in communities focusing on academic or scholarly topics. This suggests an interest in learning and knowledge-sharing within the Lithuanian online community space. The research findings align with existing literature that sees online communities as a form of civic participation, bridging the gap between individual interests and broader societal issues (Purdy, 2017; Lee, 2022). Zarifis (2019) results also showed that apart from enjoyment and entertainment, the users value the ability to reinvent yourself, convenience and institutional trust.

However, it is also evident that participation in online communities oriented towards societal issues is limited, with only 18% of surveyed internet users, or 12% of the total population, engaging with such communities. This limited engagement in social or societal matters beyond personal interests presents a significant challenge to leveraging the potential of online communities as platforms for civic participation. While these platforms can facilitate connections and conversations on broader societal issues, their effectiveness is contingent on active and diverse participation. The results suggest that these communities must do more to attract and engage users if they are to realize their full potential as forums for civic engagement. Interestingly, daily online users and those with children in their households are the most likely to visit these socially-focused online communities. This suggests that these demographics may be more inclined towards societal engagement, potentially due to their daily interaction with digital platforms or their interest in societal issues impacting their children’s futures. Despite the limited participation, those who do engage with online communities centerd on societal issues find value in the information, engaging content and diverse commentary offered. These platforms serve to broaden their perspectives and almost half of the visitors take the opportunity to voice their opinions. The topics of highest interest include ecology, environmental challenges, climate change, education and social welfare. These results are in alignment with the study of Larijani and Sravi-Moghadam (2017), which showed the culture of collectivism has huge impact on integrating.

Concerns over cybersecurity, including threats to personal data, intellectual property, and potential violations of rights and obligations, act as barriers to participation. These findings emphasize the need for online communities to prioritize the establishment of secure environments. Users need to feel safe when participating, contributing, and generating ideas that could benefit society at large. This is an area of concern in wider online community research as well. Issues surrounding data privacy and personal security have been identified as significant factors influencing individuals’ willingness to participate in online communities (Fedushko et al., 2016; Vohra and Bhardwaj, 2019). The findings of the reviewed literature also corroborate that digital communication faces two main challenges. The first relates to information security risks, such as data privacy, confidentiality and security issues. The second challenge relates to the technology that inhibits efficient and effective communication (Swart and Bond-Barnard, 2022).

Finally, results show that the platform creators should bear in mind the importance of simplicity and user-friendliness when devising technological solutions. Users of these websites express a longing for a genuine impact on socio-political issues, a valid representation for society, clarity and structure in topics, quality discourse, the freedom to voice their opinions, and user-friendly technological solutions. Interestingly, such views are prevalent across various socio-demographic user groups. One inference that can be drawn is that the advantages of virtual activities, such as openness, adaptability, and accessibility, may inadvertently contribute to additional psychological challenges for participants. The desire for user-friendly and straightforward technological solutions expressed by the users echoes the consensus in the wider research sphere that emphasizes the importance of usability in technology design. Numerous studies have found that simple, intuitive user interfaces enhance engagement and contribute to the overall success of online communities (Preece, 2001; Chou and Frank, 2018).

## 6. Conclusion

This study contributes significantly to research on online communities by providing insights into how such communities function within Lithuania and how they may evolve elsewhere. It expands our understanding of participation dynamics, demonstrating that personal interest is a key driver of active involvement. In this regard, the data indicate that online communities in Lithuania are becoming an increasingly significant channel for societal participation. They offer a more relatable and modern approach to decision-making processes and involvement in societal issues, reflecting the evolving lifestyle and communication preferences of contemporary society.

The study provides a comprehensive examination of participation dynamics within the unique context of online communities in Lithuania. It importantly highlights that personal interest serves as the central driver for active engagement, with communities focused on hobbies, lifestyle, and personal interests eliciting the highest levels of participation. Interestingly, despite the extensive adoption of online communities—observed in 63% of the surveyed Internet users—engagement in platforms focused on societal issues is limited, indicating a challenge that needs to be addressed to realize the full potential of these platforms as venues for civic participation in post-soviet countries. The study further illuminates concerns over cybersecurity, emphasizing the importance of secure environments in fostering participation and the generation of ideas for societal benefit.

The originality of the paper is national socio-cultural context of one country. However, this is also one of the limitations of this research and the reason, why the conclusions can not be generalized or applied to other countries without additional research. Another limitation is that this study relies on the answers of individual users without considering the interaction between participants. Future studies could analyze participants’ communication and interaction habits or behavioral changes over time. Results are based on a limited dataset in Lithuania. Therefore, findings can only be generalized to online communities in Lithuania. Looking forward, there are several key areas that future research can delve into. The first is the exploration of strategies to increase user engagement in online communities concentrating on societal matters. This could involve identifying and overcoming barriers to participation. Secondly, there is a need for an in-depth understanding of users’ cybersecurity concerns and an investigation into the impact of various security measures on their willingness to engage. Lastly, it would be of interest to examine the potential psychological challenges posed by participation in online communities and how these can be mitigated. For practitioners, particularly those designing online communities, several key implications arise from the findings of this study. Firstly, there’s an identified need to align community themes with potential users’ personal interests as a strategy for encouraging participation. Secondly, designers must prioritize establishing robust cybersecurity measures to build trust and create a safe environment for users. Lastly, the study underscores the importance of usability in online community design. Users expressed a strong preference for simplicity and user-friendliness in their platforms—an aspect that should not be overlooked in the design process. Taken together, the insights from this study contribute significantly to our understanding of online communities, pointing to critical areas of focus for designers and researchers alike.

## Data availability statement

The raw data supporting the conclusions of this article will be made available by the authors, without undue reservation.

## Ethics statement

Ethical review and approval was not required for the study on human participants in accordance with the local legislation and institutional requirements. Written informed consent from the patients/participants or patients/participants legal guardian/next of kin was not required to participate in this study in accordance with the national legislation and the institutional requirements.

## Author contributions

AS: Conceptualization, Methodology, Writing – original draft. MM: Conceptualization, Methodology, Writing – review & editing.
